# Body mass index, blood pressure, and glucose and lipid metabolism among permanent and fixed-term workers in the manufacturing industry: a cross-sectional study

**DOI:** 10.1186/1471-2458-14-207

**Published:** 2014-02-27

**Authors:** Mariko Inoue, Masahide Minami, Eiji Yano

**Affiliations:** 1 Graduate School of Public Health, Teikyo University, 2-11-1 Kaga, Itabashi-ku, Tokyo 173-8605, Japan; 2 Department of Public Health, School of Medicine, University of Tokyo, 7-3-1 Hongo, Bunkyo-ku, Tokyo 113-8654, Japan

**Keywords:** Job insecurity, Unstable employment, Precarious employment, Flexible work, Fixed-term workers, Temporary work, Social determinants of health, Manufacturing industry

## Abstract

**Background:**

Temporary employment, a precarious form of employment, is recognized as social determinant of poor health. However, evidence supporting precarious employment as a risk factor for health is mainly obtained from subjective data. Studies using objective clinical measurement data in the assessment of health status are limited. This study compared body mass index (BMI), lipid and glucose metabolism, and health-related lifestyle factors between permanent workers and fixed-term workers employed in the manufacturing industry.

**Methods:**

Data of 1,701 male manufacturing industry workers <50 years old in Japan were collected and analyzed. Anthropometric data were BMI, calculated using measured height and weight of study participants, and blood pressure. For lipid metabolism, low-density lipoprotein cholesterol, high-density lipoprotein cholesterol, and triglyceride levels were determined. For glucose metabolism, fasting plasma glucose and hemoglobin A1c (HbA1c) levels were measured. Multiple regression analysis adjusted for age and lifestyle factors was performed.

**Results:**

BMI was significantly higher in permanent workers (22.9 kg/m^2^) compared with fixed-term workers (22.4 kg/m^2^). The leaner population (BMI < 18.5) was greater among fixed-term workers (8.3%) compared with permanent workers (4.0%), whereas the overweight population (BMI ≥ 25.0) was greater among permanent workers (21.4%) compared with fixed-term workers (18.1%). Although fixed-term workers tended not to be overweight, regression analysis adjusted for age and lifestyle factors suggested that fixed-term employment was significantly associated with higher blood pressure (systolic *β* = 2.120, diastolic *β* = 2.793), triglyceride (*β* = 11.147), fasting blood glucose (*β* = 2.218), and HbA1c (*β* = 0.107) compared with permanent workers (all *p* < 0.01).

**Conclusions:**

Fixed-term workers showed more health risks, such as poorer blood pressure and lipid and glucose metabolism, even when adjusted for age and lifestyle variables, although BMI of fixed-term workers were lower than permanent workers. Precarious work might contribute to a deteriorating health status even among less overweight populations.

## Background

Temporary employment, a precarious form of employment [1], is recognized as a factor in health deterioration from the perspective of social determinants of health [2]. Precarious employment is categorized into four types for Japanese national statistics: (1) part-time workers, (2) temporary part-time workers ("arbeit"), (3) contract (fixed-term) workers or entrusted employees, and (4) workers dispatched by temporary labor agencies [3]. Grouping all types of precarious employment together seems inappropriate because a variety of contract types exist in the diverse occupational settings.

Fixed-term workers are employed in several industries in Japan. Around 5.6% of workers in the manufacturing industry were classified as fixed-term workers in 2011, and these workers mainly worked for heavy industry [3]. "*Kikan ko*" is the particular name for a fixed-term worker engaged in a heavy manufacturing industry, such as automobile, machine, and related manufacturing. Although fixed-term workers have been continuously involved in the manufacturing industry, their occupational health has rarely been compared with the health of permanent workers.

Workers with precarious employment (precarious workers) seem to have problems in common, such as low wages, lack of social security, and fewer rights [4]. The Japanese government has reported that the widening economic disparity may be partly attributable to increases in the number of workers with precarious employment [5]. These disadvantaged workers have some difficulty staying healthy. Previous studies have provided evidence of adverse health effects related to precarious employment [6,7], especially occupational injuries [8-10], self-rated health [11-13], suicide [14], and mental health [15]. With regard to lifestyle, workers who experienced a job change into an unstable employment situation were observed to have higher alcohol consumption at baseline levels [11], and women who changed jobs into precarious employment reduced their opportunities for physical exercise [16] in cohort studies. A cross-sectional study reported that precarious workers showed higher alcohol consumption and were more likely to miss breakfast [17].

Few studies have used objective clinical measurements to examine precarious workers’ physical health. Changes of employment status and body mass index (BMI) are the most frequently used objective measurements in cohort studies [18,19], and blood pressure was used in one Japanese cross-sectional study [17]. Use of health data from clinical laboratory tests is quite limited according to two studies that investigated associations between mental health and natural killer cells [20], and mental health and circadian cortisol levels [21]. Without objective data, we cannot capture the real health situation with regard to health risks, especially asymptomatic diseases. Subjective data might underestimate the real health risks of workers because some fixed-term workers may be afraid of providing true information for fear of losing their job. It is therefore important to measure factors using objective clinical data.

For the current study, it was hypothesized that fixed-term workers experience unstable employment and are more likely to suffer from an unhealthy lifestyle and become sick, causing a decreased quality of life. Because permanent workers can take sick leave more often than precarious workers [19], insignificant health complaints or asymptomatic conditions might go unattended by fixed-term workers. Use of clinical laboratory tests to determine asymptomatic current health status is warranted because such methods can detect the early stages of severe diseases. Based on information from previous studies, the current study compared BMI, blood pressure, lipid and glucose metabolism, and health-related lifestyle factors to investigate differences in health risks between permanent workers and fixed-term workers employed in the manufacturing industry.

## Methods

### Study participants

Study participants were manufacturing industry workers from a company in the Chubu region of Japan that employs both permanent and fixed-term workers. Permanent workers can work until their retirement age without any term limitations, whereas a fixed-term worker’s employment term with the company is 6 months. Based on observations and interviews with occupational health staff, this factory treats both permanent and fixed-term workers identically in terms of physical safety; the company provides the same protective equipment to both permanent and fixed-term workers. Based on our interviews with people from the personnel section of the company, fixed-term workers were said to experience working conditions similar to those of permanent workers, except for differences in contract length, salary, and working hours.

In the targeted company, 2,674 employees were engaged in manufacturing tasks. Of the employees in the manufacturing section, 199 (7.4%) women were excluded because of insufficient numbers for comparison with contracted workers. Additionally, 609 permanent and fixed-term workers aged 50–60 years were excluded because there were too few fixed-term workers in this age group (*n* = 33) for accurate analysis. Of the remaining 1,866 workers, 119 were excluded because anthropometric and blood pressure data were unavailable. Also excluded were 46 workers who were diagnosed with hypertension, hyperlipidemia, and diabetes. Data from 1,701 workers were analyzed.

### Health data

Data were collected from the 2008 annual health check-up. The annual health check-up is mandated by the Industrial Safety and Health Law in Japan. Anthropometric data and blood pressure, lipid and glucose metabolism, and health-related lifestyle data were collected.

Anthropometric data included height, weight, and BMI calculated using measured height and weight of participants. Blood pressure, both systolic and diastolic, was measured with a Parama-Tech UM-15P with attendance of skilled occupational health nurses. For lipid metabolism, low-density lipoprotein cholesterol (LDL-cholesterol), high-density lipoprotein cholesterol (HDL-cholesterol), and triglyceride levels were examined. For glucose metabolism, fasting plasma glucose and hemoglobin A1c (HbA1c) levels were measured.

### Health-related lifestyle data

Health-related lifestyle data were obtained from a self-administered questionnaire conducted during the annual health check-up.

Smoking status was determined by answers to the question: "Please choose your smoking status." Respondents chose one of three categories (non-smoker, ex-smoker, or current smoker). Only current smokers were included in the "smoker" group. Alcohol consumption was determined by answers to the question: "Please choose one category regarding drinking behavior." Respondents chose either "drinking" or "almost no drinking". Respondents who chose "drinking" then reported how many days per week and the amount of alcohol consumed. A scale of alcohol consumption, i.e., how many glasses of beer, wine, whiskey, or Japanese hard liquor, were equal to one go of sake, was provided. Those who drank more than 198 g of ethanol per week were categorized as heavy drinkers. This threshold was based on the unit of measurement of sake (Japanese rice wine), called "*go*" in Japanese. One go of sake contains 22 g of ethanol. Study participants who drank more than 1.5 go of sake 6 days per week or more were determined to be heavy drinkers Frequency of exercise was determined by answers to the question: "Did you exercise more than twice per week this year?" Exercise was defined as more than 30 minutes of physical activity at a time. Answers were grouped into "yes" or "no". This style of questioning is usually used in annual health check-ups in Japan, and also in the National Health and Nutrition Survey by the Ministry of Health, Labour and Welfare of Japan, and other previous studies [22,23].

Questions on other aspects of lifestyle required yes or no answers. Breakfast-eating habits were determined by answers to the question: "Do you have breakfast more than 4 days per week?" Time dinner was eaten was determined by answers to the question: "Do you eat dinner within 2 hours of going to bed more than 3 days per week?" Sleep satisfaction was determined by answers to the question: "Do you feel rested after sleep?" Overtime hours of work were determined by answers to the question: "Do you work overtime more than 2 hours per day on average?"

### Statistical analysis

Age, anthropometry, blood pressure, lipid and glucose metabolism, and health-related lifestyle data were descriptively analyzed. Data were then compared between permanent and fixed-term workers. Age comparisons were tested using the Wilcoxon rank-sum test because of data distribution. The proportion of participants in each of the health-related lifestyle categories were compared using the Cochran–Mantel–Haenszel test, adjusting for one-year age class. Analysis of covariance was performed for anthropometry, blood pressure, and lipid and glucose metabolism data between permanent and fixed-term workers, controlled for the effects of age. Multiple regression analysis was used to estimate differences between permanent and fixed-term workers with respect to BMI, blood pressure, and lipid and glucose metabolism data. Model 1 was adjusted for age; and Model 2 was adjusted for age, smoking, alcohol consumption, and exercise behavior. Because some of our data on risk factors were not normally distributed, we took the logarithm for our multi-regression analysis.

### Ethical considerations

The Institutional Review Board of the Teikyo University School of Medicine approved the study design (No. 10-114). Because this observational study did not use human biological specimens and used only existing data, participant informed consent was not necessary according to the Ethical Guidelines for Epidemiological Research determined by the government [24].

## Results

Participants were 1,701 male factory workers. Their basic characteristics, including anthropometric data, blood pressure, lipid and glucose metabolism, and health-related lifestyle factors, are shown in Table 1. Mean BMI, blood pressure, lipid metabolism, and glucose metabolism were within normal ranges.

**Table 1:** Basic characteristics of the study population

**Variables**	**N = 1,701**		
Age (mean, SD)	30.7	(8.0)	
Job type n (%)			
Permanent	1,363	(80.1%)	
Fixed-term	338	(19.9%)	
Health related lifestyle n (%)			
Current smoker	1,001	(58.9%)	
Alcohol consumption, yes	830	(48.8%)	
Heavy drinker	211	(12.4%)	
Doing exercise, yes	300	(17.6%)	
Eating breakfast	1,192	(70.1%)	
Eating dinner late	377	(22.2%)	
Sleep satisfaction, good	849	(49.9%)	
Overtime work	890	(52.3%)	
Anthropometric data (mean, SD)			
Height (cm)	172.3	(5.6)	
Weight (kg)	67.8	(10.8)	
Body mass index (kg/m^2^)	22.8	(3.3)	
BMI category n (%)			
Obese (BMI ≥ 25.0)	353	(20.8%)	
Lean (BMI < 18.5)	83	(4.9%)	
Blood pressure (mean, SD)			
Systolic (mmHg)	122.8	(13.6)	
Diastolic (mmHg)	70.0	(11.6)	
Lipid metabolism (mean, SD)			
LDL-cholesterol (mg/dL)	112.6	(31.6)	
HDL-cholesterol (mg/dL)	63.5	(14.7)	
Triglyceride (mg/dL)	90.4	(65.5)	
Glucose metabolism (mean, SD)			
Fasting plasma glucose (mg/dL)	88.4	(10.2)	
HbA1c (%)	4.9	(0.3)	

SD: standard deviation, BMI: body mass index.

Definition

Alcohol consumption: Persons who answered "yes" for the question "Do you drink alcohol?".

Heavy drinker: Persons who drink alcohol more than 198 g of ethanol per week.

Doing exercise: Persons who answered "yes" for the question, "Have you exercised more than twice a week for more than one year?".

For health-related lifestyle categories, the proportion of workers reporting heavy drinking, eating dinner late, working overtime, and not exercising was higher in permanent workers than in fixed-term workers. The proportion of current smokers was higher in fixed-term workers than in permanent workers. No differences in breakfast-eating habits and sleep satisfaction between permanent and fixed-term workers were found (Table 2).

**Table 2:** Comparison of age and percentage of health-related lifestyle of permanent and fixed-term workers

**Variables**	**Permanent**		**Fixed-term**		***p*-value**	
	**(n = 1,363)**		**(n = 338)**			
Age mean (standard deviation)	30.9	(8.0)	29.6	(7.9)	0.003	**
Health related lifestyle n (%)						
Current smoker	786	(57.7%)	215	(63.6%)	0.027	*
Alcohol consumption, yes	676	(49.6%)	154	(45.6%)	0.422	
Heavy drinker	189	(13.9%)	22	(6.5%)	0.002	**
Doing exercise, yes	223	(16.4%)	77	(22.8%)	0.009	**
Eating breakfast	946	(69.4%)	246	(72.8%)	0.313	
Eating dinner late	324	(23.8%)	53	(15.7%)	0.004	**
Sleep satisfaction, good	670	(49.2%)	179	(53.0%)	0.341	
Overtime work	768	(56.4%)	122	(36.1%)	<0.001	**

Wilcoxon rank sum test was used to compare age between permanent and fixed-term workers.

Cochran-Mantel-Haenszel test was used to compare health related lifestyle between permanent and fixed-term workers. *p < 0.05 **p < 0.01.

BMI and lipid and glucose metabolism were compared by contract type (Table 3). The BMI of permanent workers was higher than that of fixed-term workers, even after adjusting for age. However, blood pressure, triglyceride, fasting blood glucose, and HbA1c values were higher in fixed-term workers than in permanent workers.

**Table 3:** Comparison of anthropometric data, blood pressure, and metabolism levels of permanent and fixed-term workers

**Variables**	**Permanent**		**Fixed-term**		***p*-value**		
	**(n = 1,363)**		**(n = 338)**				
Anthropometric data (mean, SD)							
Height (cm)	172.5	(0.2)	171.8	(0.3)	0.056		
Weight (kg)	68.2	(0.3)	66.2	(0.6)	0.002	*	
Body mass index (kg/m^2^)	22.9	(0.1)	22.4	(0.2)	0.012	**	
Obese (BMI ≥ 25.0) n(%)	292	(21.4%)	61	(18.1%)	0.340		
Lean (BMI < 18.5) n(%)	55	(4.0%)	28	(8.3%)	0.003	*	
Blood pressure (mean, SD)							
Systolic (mmHg)	122.4	(0.4)	124.4	(0.7)	0.013	*	
Diastolic (mmHg)	69.5	(0.3)	72.1	(0.6)	<0.001	**	
Lipid metabolism (mean, SD)							
LDL-cholesterol (mg/dL)	113.1	(0.8)	110.3	(1.6)	0.124		
HDL-cholesterol (mg/dL)	63.7	(0.4)	62.6	(0.8)	0.195		
Triglyceride (mg/dL)	88.2	(1.8)	99.1	(3.5)	0.006	**	
Glucose metabolism (mean, SD)							
Fasting plasma glucose(mg/dL)	88.1	(0.3)	90.2	(0.8)	0.012	*	
HbA1c (%)	4.8	(0.01)	5.0	(0.02)	<0.001	**	

SD: standard deviation, BMI: body mass index.

Analysis of covariance was used to compare the mean of each data between permanent and fixed-term workers.

Age adjusted least square mean was shown in the Table. *p < 0.05 **p < 0.01.

Cochran-Mantel-Haenszel test was used to compare the proportion of BMI categories between permanent and fixed-term workers. **p < 0.01.

Table 4 shows the results of linear regression models used to examine BMI, blood pressure, and lipid and glucose metabolism relationships between contract types. Fixed-term employment was significantly associated with a lower BMI, and higher blood pressure, triglyceride, fasting blood glucose, and HbA1c values, even after controlling for the effects of age and lifestyle.

**Table 4:** Regression model showing association of precarious employment and body mass index, blood pressure, lipid and glucose metabolism

**Variables**	**Model 1**				**Model 2**			
	**Parameter estimate**	**Standard error**	**Standardized estimate**	***p*-value**	**Parameter estimate**	**Standard error**	**Standardized estimate**	***p*-value**
Anthropometric data								
Body mass index (kg/m^2^)	-0.504	0.201	-0.060	0.005	-0.471	0.201	-0.056	0.008
Blood pressure								
Systolic (mmHg)	2.044	0.826	0.060	0.008	2.120	0.827	0.062	0.006
Diastolic (mmHg)	2.574	0.641	0.088	<0.001	2.793	0.638	0.096	<0.001
Lipid metabolism								
LDL-cholesterol (mg/dL)	-2.784	1.809	-0.035	0.168	-2.485	1.803	-0.031	0.224
HDL-cholesterol (mg/dL)	-1.162	0.896	-0.031	0.087	-0.948	0.878	-0.026	0.132
Triglyceride (mg/dL)	10.902	3.938	0.066	0.004	11.147	3.943	0.068	0.004
Glucose metabolism								
Fasting plasma glucose (mg/dL)	2.161	0.856	0.072	0.006	2.218	0.857	0.074	0.004
HbA1c (%)	0.110	0.022	0.128	<0.001	0.107	0.022	0.124	<0.001

Model 1 was adjusted for age.

Model 2 was adjusted for age, smoking, alcohol, and exercise.

Results of *p*-value is calculated by the regression analysis with use of log-transformed data.

## Discussion

Fixed-term workers had poorer blood pressure, triglyceride, fasting blood glucose, and HbA1c values than those of permanent workers after controlling for the effects of age and lifestyle; although fixed-term workers had lower average BMI. The results partially supported our hypothesis that fixed-term workers have a poorer health status than permanent workers.

Our hypothesis that fixed-term workers would show worse lifestyles factors than permanent workers was found to be correct for smoking only in our study population. The results of the current study align with other studies which have reported that the smoking rate was higher among precarious workers [13]. Our study results showed that fixed-term workers with lower BMI had higher blood pressure, and their triglyceride levels were higher based. A previous study concluded that smoking was related to high blood pressure and diabetes [25], and another study reported that a population of leaner individuals who smoked showed a higher mortality rate from cardiovascular diseases [26]. Regardless of employment type, the smoking rate of the current study’s participants (58.6%) was higher than the national average (47.3%) for the same age group in 2008 [27]. The smoking rate of Japanese workers varies by occupation. Compared with professional workers, workers who are engaged in production, construction, or manufacturing are 1.5 times more likely to smoke [28]. This finding applied to the current study’s population. Thus, smoking may be tied not to employment type but, rather, to the industry in which an individual is employed. However, fixed-term workers in the current study had higher rates of smoking than permanent workers.

Contrary to the study’s hypothesis, fixed-term workers seemed to have healthier lifestyles than permanent workers in terms of diet and physical exercise. Permanent workers appeared to have difficulty maintaining a healthy lifestyle. The percentage of heavy alcohol consumption, lack of exercise, late-night eating habits, and overtime work was higher among permanent workers when compared with fixed-term workers. Failing to exercise and eating dinner late were characteristic of permanent workers. These patterns may be a result of overtime work. Because 52.2% of permanent workers consistently work overtime (compared with 35.7% of fixed-term workers), excess working hours might deprive permanent workers of any free time. This may be influenced by contract type. Permanent workers often work hard and feel more pressure from their workload [29]. The results of the current study also suggest that permanent workers are at greater risk for chronic disease because of stress in their jobs and their lifestyles.

It is possible that fixed-term work might contribute to a lower socioeconomic status, as suggested by the Japanese government. Generally, precarious workers have lower salaries than permanent workers in Japan, with permanent workers earning 2.5 times more than precarious workers [30]. There is, therefore, a gap in socioeconomic status between permanent and precarious workers. A previous study reported that diabetes was associated with a lower socioeconomic status [31]. Food preference might be one cause of high blood pressure. Previous studies have reported that a lower-income population has a more unhealthy diet and consumes more take-out food. Dietary habits often differ according to socioeconomic status [32]. Unhealthy food choices associated with a low-income population could yield the same results for fixed-term workers who earn lower wages when compared with permanent workers. The current study only examined the frequency of meals, and did not look at content. The data, therefore, do not provide information on the content of food consumed, so no conclusion can be reached on this point.

Some particular occupational conditions experienced by fixed-term workers, such as repeated exposure to new occupational environments because of unstable job contracts, might influence their health risks. Previous studies have reported that high blood pressure was associated with job insecurity [33]. Although fixed-term workers are subject to job insecurity, they are still healthy and can work. This healthy-worker effect might influence our study results.

This study had several limitations. One hundred and nineteen employees who did not provide anthropometric data were excluded. It is possible that this missing healthy population might have decided not to attend check-ups because they thought, and possibly correctly, that they were healthy.

Only permanent workers can be a chief of an assembly line or hold management positions in the factory. This difference might be a confounding variable in the analysis because it could influence employment status and health outcomes. Mental pressure in permanent workers may increase as they seek to be promoted, while fixed-term workers have no chance of promotion. This may negatively impact workers’ health because of a feeling of anxiety or pressure to change jobs. These differences are concordant with previous studies [34].

In terms of workers’ backgrounds, socioeconomic status and family factors were not determined in detail in the current study. Marital status or having children might influence workers’ lifestyles (especially diet) because family support can create healthier behaviors and eating habits. National statistics reveal that 32.2% of single permanent workers married, while 17.2% of single workers with precarious employment married after a 6-year follow-up [35]. The rate of marriage in the current study population may differ by contract type. It is possible that working men who are married have a more stable and nutritionally balanced diet. With regard to education, the company employs high school graduate-level workers in the factory regardless of contract type. The percentage of residents who attended high school in Japan has been maintained at 95% over the past two decades [36]. Thus, educational levels of participants were likely to be similar.

The working period of permanent workers was not determined during the study. Therefore, young permanent workers in the participant population included those who started work only recently. The youngest permanent workers in the study population were 20 years old. These workers should have less work experience. These permanent workers might share experiences similar to those of fixed-term workers. Also, questions about fixed-term workers’ previous work experiences were not asked, nor was career trajectory taken into account. Some may have been fixed-term employees from the beginning, whereas others may have been employed as permanent workers previously and changed to fixed-term at a later date. Career trajectories, which might influence workers’ health [11,16], were not investigated. Finally, the study was a cross-sectional study; the results cannot provide causal relationships between precarious employment and health risks.

## Conclusion

This study evaluated the health risks of fixed-term workers in the manufacturing industry in Japan using objective measurements. The results of the analysis were adjusted for lifestyle variables, and associations between fixed-term work and greater health risks in terms of blood pressure and lipid and glucose metabolism were found. In contrast, permanent workers had better blood pressure and lipid and glucose metabolism, but had difficulty maintaining a healthy lifestyle in terms of diet, alcohol consumption, and exercising, which may be because of overwork. Our hypothesis that only precarious workers have deteriorating health was partially correct, with fixed-term workers also showing health risks although they were less likely to be overweight. This suggests that caution should be taken even for those with lower BMI and may be asymptomatic. Further research is required using cohort studies to investigate causal relationships between precarious employment and health risks.

## Competing interests

The authors declare that they have no competing interests.

## Authors’ contributions

MI planned the study, analyzed data, and drafted the manuscript. MM contributed to obtaining data from the target population. All authors participated in the design and coordination of the study. All authors read and approved the final manuscript for submission.

## Pre-publication history

The pre-publication history for this paper can be accessed here:

http://www.biomedcentral.com/1471-2458/14/207/prepub
